# A network pharmacology approach to explore the mechanisms of Erxian decoction in polycystic ovary syndrome

**DOI:** 10.1186/s13020-018-0201-1

**Published:** 2018-08-29

**Authors:** Lihong Liu, Bo Du, Haiying Zhang, Xiaofei Guo, Zheng Zhou, Aihui Xiu, Chang Liu, Shiyu Su, Hao Ai

**Affiliations:** 1 Department of Gynecological Ward, The Third Affiliated Hospital, Jinzhou Medical University, Jinzhou, China; 2 Liaoning Provincial Key Laboratory of Follicle Development and Reproductive Health (Office of Science and Technology), Jinzhou, China; 3 Library Department, Jinzhou Medical University, Jinzhou, China; 4 Department of Gynecological Ward, The First Affiliated Hospital, Jinzhou Medical University, Jinzhou, China

**Keywords:** System pharmacology, Erxian decoction, Polycystic ovary syndrome, Pharmacological mechanism, Targets

## Abstract

**Background:**

Polycystic ovary syndrome (PCOS) significantly affects women’s health and well-being. To explore the pharmacological basis of the Erxian decoction (EXD) action in PCOS therapy, a network interaction analysis was conducted at the molecular level.

**Methods:**

The active elements of EXD were identified according to the oral bioavailability and drug-likeness filters from three databases: traditional Chinese medicine system pharmacology analysis platform, TCM@taiwan and TCMID, and their potential targets were also identified. Genes associated with PCOS and established protein–protein interaction networks were mined from the NCBI database. Finally, significant pathways and functions of these networks were identified using Gene Ontology and Kyoto Encyclopedia of Genes and Genomes analyses to determine the mechanism of action of EXD.

**Results:**

Seventy active compounds were obtained from 981 ingredients present in the EXD decoction, corresponding to 247 targets. In addition, 262 genes were found to be closely related with PCOS, of which 50 overlapped with EXD and were thus considered therapeutically relevant. Pathway enrichment analysis identified PI3k-Akt, insulin resistance, Toll-like receptor, MAPK and AGE-RAGE from a total of 15 significant pathways in PCOS and its treatment.

**Conclusions:**

EXD can effectively improve the symptoms of PCOS and our systemic pharmacological analysis lays the experimental foundation for further clinical applications of EXD.

## Background

Polycystic ovary syndrome (PCOS) affects 5–20% of all reproductive aged women around the world, and is characterized by hyper-androgenism, infertility, irregular menstrual cycle and polycystic ovarian morphology (PCOM) due to abnormal production of androgens by the ovaries [1]. In addition, metabolic disruptions like hyperinsulinemia and abnormal adipokine secretion from the adipose tissue is also seen [2]. PCOS not only compromises women’s physical and mental health, but also increases the risk of type 2 diabetes mellitus (T2DM), atherosclerosis, cardiovascular disease, endometrial cancer, breast cancer and other long-term complications. Currently, PCOS treatment mostly relies on anti-androgen drugs, insulin sensitizers, and ovulation-promoting drugs [3]. Studies show that PCOS is frequently associated with insulin signaling [4], PI3K-Akt Signaling Pathway [5], FoxO 1 Signaling [6], and non-alcoholic fatty liver disease (NAFLD) [7], but the underlying mechanisms are not clear.

**Fig. 1:**
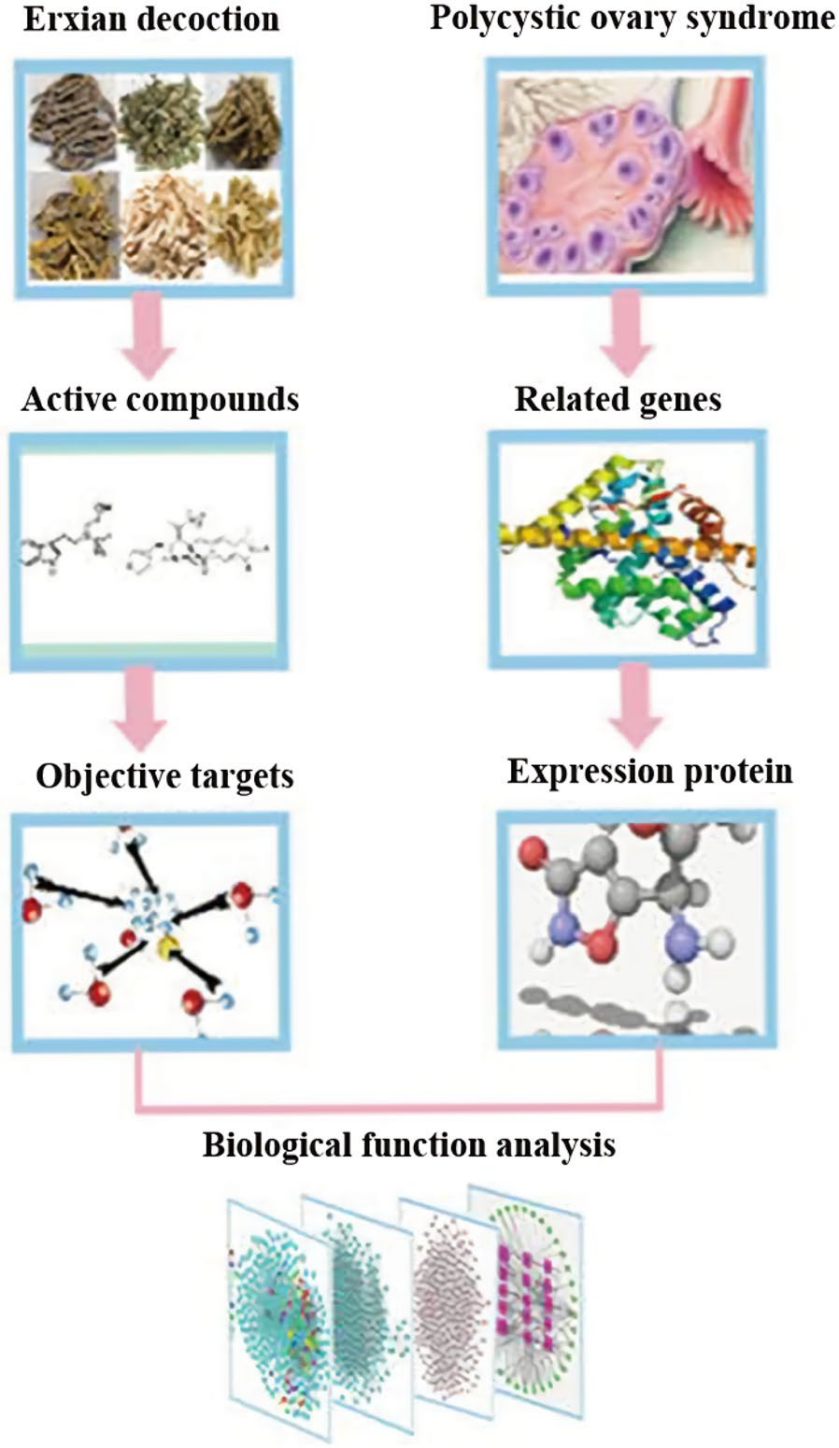
A schematic diagram of the systems biology-based methodologies for unraveling the pharmacological basis of EXD action in PCOS

Traditional Chinese medicine (TCM) has been continually practiced since 2000 years. The Erxian decoction (EXD) consists of six herbs: *Epimedium brevicornum* (Yinyanghuo), *Curculigo orchioides* (Xianmao), *Morinda officinalis* (Bajitian), *Angelica sinensis* (Danggui), *Anemarrhena asphodeloides* (Zhimu) and *Phellodendron chinense* (Huangbo), and is used to mitigate menopausal side effects [8], osteoporosis [9], and ovarian failure [10]. One study showed that EXD upregulated estrogen receptor, enhanced ovarian function, reduced serum FSH and LH levels, increased E2 and progesterone levels, decreased malonic dialdehyde (MDA) in ovarian tissues, increased total anti-oxidative capacity (T-AOC), reduced follicular atresia by increasing follicle count, and reduced cell stromal hyperplasia [11]. Another study found that EXD could restore menstrual cycle, regulate hypothalamic-pituitary-ovarian axis function, increase steroid hormone secretion, restore primordial follicle recruitment and superior follicle selection, and improve ovulation rate and ovarian function [12]. Although EXD has been used clinically for gynecological diseases for more than 60 years, its mechanism of action is unclear due to its complex composition. In order to enhance their therapeutic efficacy, it is essential to elucidate the molecular and biological basis of TCM preparations. Systems pharmacology (SP) has recently emerged as a technique to decipher complex pharmacological problems [13]. Recently, the traditional Chinese medicine systems pharmacology database and analysis platform (TCMSP) was developed as a digital repository of traditional medicines. In addition, it can predict pharmacological targets and specific maladies of every dynamic compound, and is a major analytical tool in network pharmacology that helps determine the complex interactions between drugs and targets [14]. Since TCM formulations have multiple targets and complex ingredients, SP can even predict novel compounds based on existing formulations [15]. Network pharmacology has helped elucidate the mechanism of several TCM formulations so far [16, 17]. In this study, we used the SP approach to determine the potential mechanism of EXD action in treating PCOS. We first screened the TCMSP database for active compounds of EXD and identified its targets, followed by mining for disease-related genes, and network analysis of those genes (Fig. 1).

## Methods

### Identification of active EXD compounds

TCMSP (http://lsp.nwu.edu.cn/tcmsp.php) is a Chinese medicine pharmacology database containing information about the herbs used in TCM, and absorption, distribution, metabolism and excretion (ADME) characteristics of the individual compounds, their targets, related diseases, and pathways. The database search for EXD revealed its constituent herbs (*E. brevicornum, C. orchioides, M. officinalis, A. sinensis, A. asphodeloides, P. chinense*). In drug research and development, approximately 90% of the novel candidates fail the tests due to unexpected toxicity, poor absorption or bioavailability (in addition to other biopharmaceutical/metabolic issues), or poor efficacy [18]. Although the drug ADME characteristics are highly significant, biological testing of each and every candidate drug is impractical due to the high-costs and time involved. In recent years, the focus has therefore shifted to bioinformatics to determine the pharmacokinetic properties of candidate drugs. In order to maximize the chances of finding the fully active compounds, we set two conditions as the criteria for screening these active compounds—oral bioavailability (OB) and drug-likeness (DL), which are the two most important indicators for evaluating ADME characteristics via bioinformatics.

Orally administered drugs must pass some obstructions, like P-glycoprotein (P-gp) [19] and cytochrome P450 s [20], before reaching their target site. The OB of candidate drugs can be predicted with QSAR modeling using linear [multiple linear regression (MLR) and partial least squares regression (PLS)] and nonlinear [support-vector machine regression (SVR)] methods, and the OBioavail 1.2 program [21]. The compounds with OB ≥ 30% were filtered for further analysis. DL, i.e. similarities with the physiochemical or/and structural properties of existing drugs is used to filter out compounds with undesirable qualities [22]. The Dragon program was used to determine the DL index based on parameters like molecular weight, one-dimensional descriptors (e.g. logP, H-donors and H-acceptors), two-dimensional profiles (e.g. extremity number, worldwide topological charge file), three-dimensional variables (average geometric distance degree and radius of gyration), and total positive and negative charges. The DL index of any new molecule is calculated based on Tanimoto similarity [23] as per the formula:

$$f(A,\;B) = \frac{A \cdot B}{{\left| A \right|^{2} + \left| B \right|^{2} - A \cdot B}}$$

where A represents the descriptor of the new numerator and B represents all the 6511 molecules selected from the Drug Bank database. The average of all descriptors was calculated by Dragon and the compounds with DL index ≥ 0.18 were selected.

### Prediction of EXD targets and compound-target network establishment

An essential step following the discovery of active molecules is to identify their molecular targets that trigger the biological effects [24]. Bioinformatics methodologies like chemometrics and chemogenomics are often used to mine and integrate information, in order to identify the molecular targets [25]. The Chinese chemical databases and PubChem were mined for the compounds, and their physicochemical properties and biological targets were determined. The genetic information of the targets were obtained from the UniProt Online Resources (http://www.uniprot.org) [26]. Random Forest and Support Vector Machine (SVM) method and prediction models were used for large scale chemical simulation of the drug targets [27, 28]. A compound-target network refers to a mathematical and computable expression of different associations between TCM formulae and diseases, especially in complex natural frameworks [29]. Target interactions were obtained from the STITCH protein database (http://stitch.embl.de/) [30]. The relationship between the above candidate compounds and potential targets were determined with EXCEL as the input source, and the Cytoscape program was used to form a compound-target visual interaction network (CT network) [31]. The nodes in the network are compounds, proteins, enzymes, and targets, and the relationship between them is represented by the lines between the nodes [32].

### PCOS-EXD gene network establishment

Genes related to PCOS were downloaded from the NCBI Gene database (http://www.ncbi.nlm.nih.gov/quality) [33]. The database was searched using the keyword ‘Polycystic ovary syndrome’, which yielded 296 known PCOS-related genes of *Homo sapiens*. The CT network was then mapped to the PCOS-related gene network to establish a PCOS-drug interaction network based on overlapping genes, to determine the mechanism of EXD action in PCOS. In such a network, a node can represent a herb, a compound, or a gene/protein, and an “edge” is an association between the nodes. The “degree” of a hub is the number of edges associated with it, and the “betweenness” of a hub is the number of closest associations. The nodes whose connectivity was greater than twice the median of all nodes are selected as the hub nodes in the network. The hubs with high centrality are considered the key hubs in a network.

### Biological function analysis

Gene Ontology (GO, http://www.geneontology.org/) and Kyoto Encyclopedia of Genes and Genomes (KEGG, http://www.genome.jp/kegg/) pathway analysis were used to analyze the primary pharmacological units. GO is a database that functionally annotates genes and proteins into three main terms—cellular components (CC), molecular functions (MF), and biological processes (BP) [34] and pathway analysis reveals the possible biological processes with key hub genes. KEGG is a database for determining the high-level functions and biological relevance of a large set of genes [35]. The molecular action of mechanism of EXD could be elucidated by analyzing the significant GO terms and pathways of the overlapping genes. The ClueGO plugin of Cytoscape was used to integrate the GO terms with KEGG pathways [36].

## Results

### OB prediction and DL calculation

**Fig. 2:**
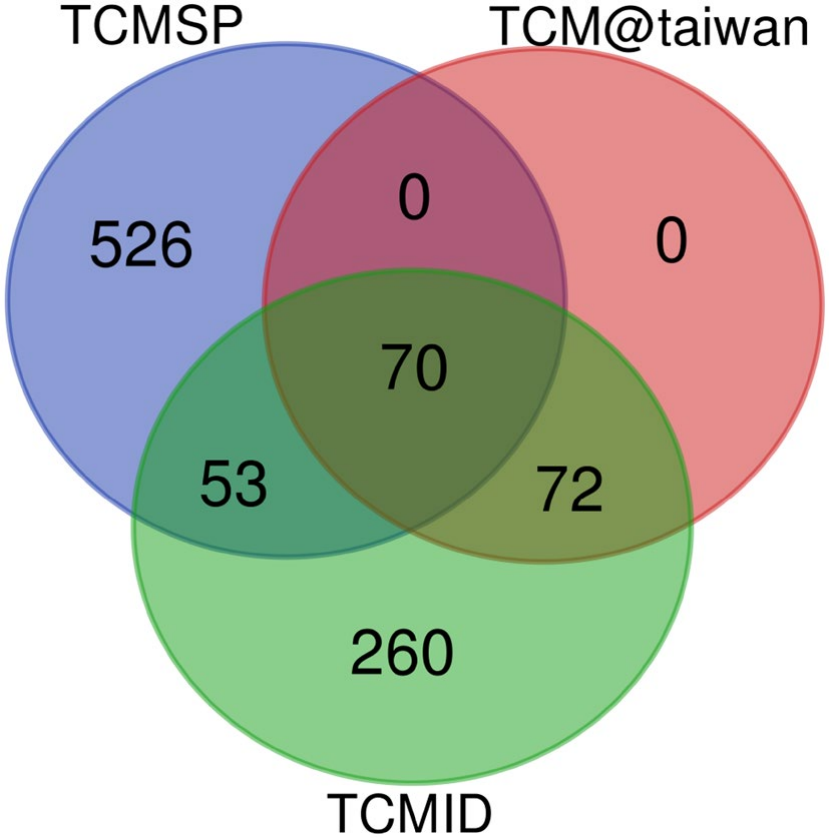
A total of 981 compounds were obtained from three databases, including 649 from TCMSP, 142 from TCM@taiwan and 455 from TCMID, of which 70 compounds were shared across all databases

We obtained 981 active compounds from the initial search of three databases: 649 from TCMSP, 142 from TCM@taiwan, and 455 from TCMID. There were 123 common compounds between the TCMSP and TCMID databases, while TCM@taiwan and TCMID had 142 common compounds amongst them, and 70 compounds were shared across the three databases (Fig. 2). Seventy compounds passed the OB and DL filters and had favorable pharmacokinetic profiles, and a total of 247 targets were identified for these compounds. The pharmacokinetic properties of the compounds and the corresponding number of targets are shown in Table 1.

**Table 1:** Active compounds properties

Compound	Herb	Chemical	OB	DL	Degree
C01	CR01	ZINC03982454	36.91	0.76	2
C02	CR02	Cycloartenol	38.69	0.78	1
C03	CR03 MO13 AS01 PC14	Beta-sitosterol	36.91	0.75	38
C04	CR04	3,2′,4′,6′-Tetrahydroxy-4,3′-dimethoxy chalcone	52.69	0.28	11
C05	CR05	Curculigoside B_qt	83.36	0.19	5
C06	CR06 AS02 AR07 PC10	Stigmasterol	43.83	0.76	31
C07	EH01	24-Epicampesterol	37.58	0.71	2
C08	EH02	Linoleyl acetate	42.1	0.2	4
C09	EH03 PC22	Poriferast-5-en-3beta-ol	36.91	0.75	2
C10	EH04	DFV	32.76	0.18	11
C11	EH05	Chryseriol	35.85	0.27	18
C12	EH06 MO12	Sitosterol	36.91	0.75	3
C13	EH07 AR02	Kaempferol	41.88	0.24	63
C14	EH08	Olivil	62.23	0.41	4
C15	EH09 AR03	Anhydroicaritin	45.41	0.44	37
C16	EH10	C-Homoerythrinan,1,6-didehydro-3,15,16-trimethoxy-,(3.beta.)-	39.14	0.49	38
C17	EH11	Yinyanghuo A	56.96	0.77	9
C18	EH12	Yinyanghuo C	45.67	0.5	11
C19	EH13	Yinyanghuo E	51.63	0.55	11
C20	EH14	6-Hydroxy-11,12-dimethoxy-2,2-dimethyl-1,8-dioxo-2,3,4,8-tetrahydro-1H-isochromeno[3,4-h]isoquinolin-2-ium	60.64	0.66	6
C21	EH15	8-(3-Methylbut-2-enyl)-2-phenyl-chromone	48.54	0.25	30
C22	EH16	Anhydroicaritin-3-O-alpha-L-rhamnoside	41.58	0.61	1
C23	EH17	1,2-bis(4-Hydroxy-3-methoxyphenyl)propan-1,3-diol	52.31	0.22	11
C24	EH18	Icariin	41.58	0.61	1
C25	EH19	Icariside A7	31.91	0.86	3
C26	EH20	Luteolin	36.16	0.25	57
C27	EH21 PC15	Magnograndiolide	63.71	0.19	4
C28	EH22 PC19	Quercetin	46.43	0.28	153
C29	MO01	Ethyl oleate (NF)	32.4	0.19	1
C30	MO02	Alizarin-2-methylether	32.81	0.21	13
C31	MO03	1-Hydroxy-6-hydroxymethylanthracenequinone	81.77	0.21	12
C32	MO04	(2R,3S)-(+)-3′,5-Dihydroxy-4,7-dimethoxydihydroflavonol	77.24	0.33	9
C33	MO05	1,6-Dihydroxy-5-methoxy-2-(methoxymethyl)-9,10-anthraquinone	104.54	0.34	12
C34	MO06	Americanin A	46.71	0.35	11
C35	MO07	2-Hydroxy-1,8-dimethoxy-7-methoxymethylanthracenequinone	112.3	0.37	11
C36	MO08	2-Hydroxy-1,5-dimethoxy-6-(methoxymethyl)-9,10-anthraquinone	95.85	0.37	14
C37	MO09	1,5,7-Trihydroxy-6-methoxy-2-methoxymethylanthracenequinone	80.42	0.38	10
C38	MO10	Diop	43.59	0.39	3
C39	MO11	3beta,20(R),5-alkenyl-stigmastol	36.91	0.75	1
C40	MO14	Isoprincepin	49.12	0.77	2
C41	MO15	3beta-24S(R)-butyl-5-alkenyl-cholestol	35.35	0.82	1
C42	MO16	Ohioensin-A	38.13	0.76	3
C43	AR01	Asperglaucide	58.02	0.52	5
C44	AR04	Anemarsaponin F_qt	60.06	0.79	1
C45	AR05	Hippeastrine	51.65	0.62	11
C46	AR06	Timosaponin B III_qt	35.26	0.87	2
C47	AR08	Icariin I	41.58	0.61	1
C48	AR09	(Z)-3-(4-hydroxy-3-methoxy-phenyl)-N-[2-(4-hydroxyphenyl)ethyl]acrylamide	118.35	0.26	8
C49	AR10	Diosgenin	80.88	0.81	16
C50	AR11	Coumaroyltyramine	112.9	0.2	10
C51	PC01	Berberine	36.86	0.78	17
C52	PC02	Coptisine	30.67	0.86	9
C53	PC03	Phellavin_qt	35.86	0.44	3
C54	PC04	Delta 7-stigmastenol	37.42	0.75	1
C55	PC05	Phellopterin	40.19	0.28	12
C56	PC06	Dehydrotanshinone II A	43.76	0.4	21
C57	PC07	Rutaecarpine	40.3	0.6	18
C58	PC08	Skimmianin	40.14	0.2	5
C59	PC09	Chelerythrine	34.18	0.78	6
C60	PC11	Worenine	45.83	0.87	7
C61	PC12	Cavidine	35.64	0.81	28
C62	PC13	Hericenone H	39	0.63	1
C63	PC16	Palmatine	64.6	0.65	19
C64	PC17	Fumarine	59.26	0.83	28
C65	PC18	Isocorypalmine	35.77	0.59	36
C66	PC20	Phellamurin_qt	56.6	0.39	10
C67	PC21	(*S*)-Canadine	53.83	0.77	32
C68	PC23	Berberrubine	35.74	0.73	13
C69	PC24	Campesterol	37.58	0.71	1
C70	PC25	Thalifendine	44.41	0.73	14

### Network analysis

**Fig. 3:**
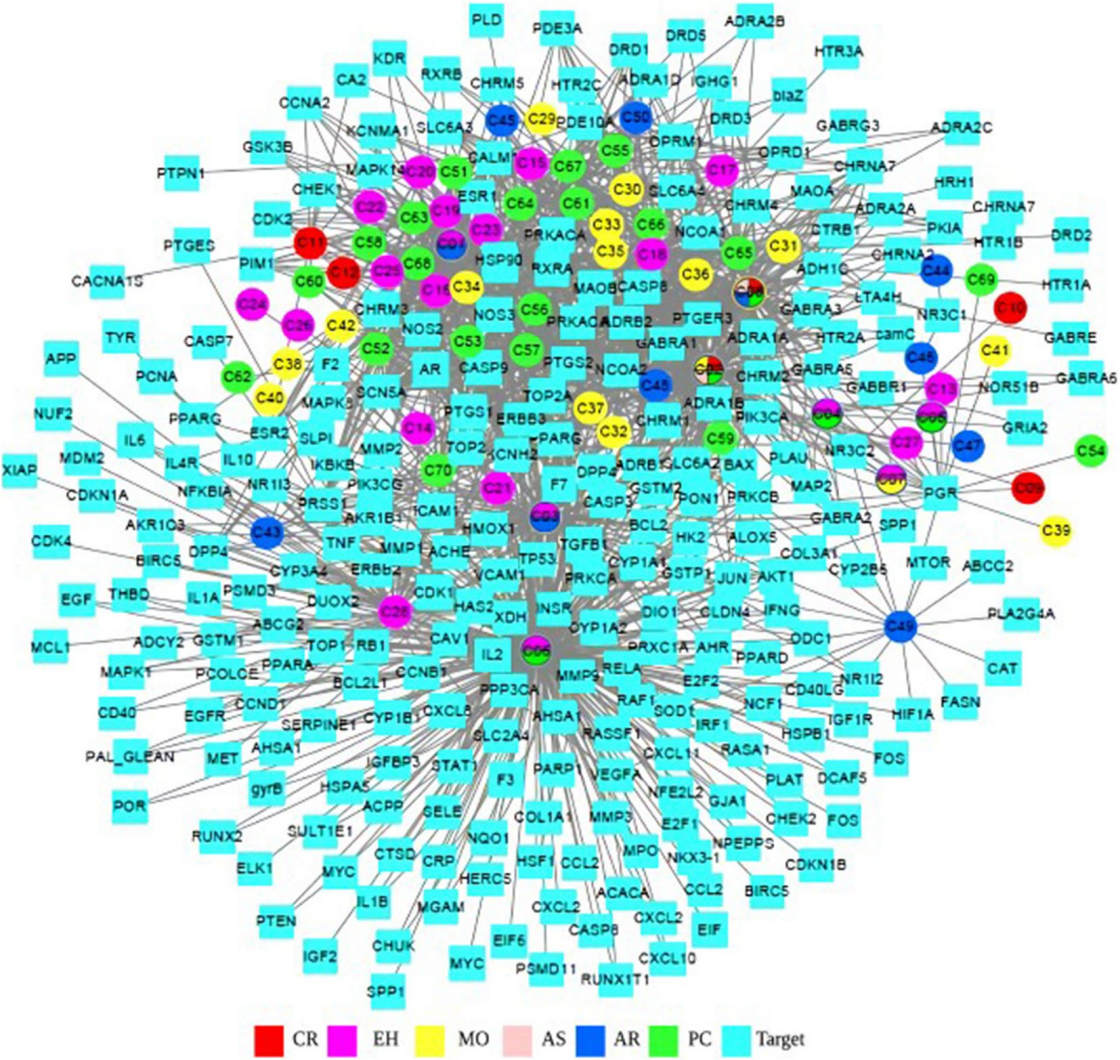
C-T network, The multi-colored circles represent different herbs and squares represent the targets. (red for *Curculigo*, dark pink for *Epimedium*, yellow for *Morinda*, light pink for *Angelica*, dark blue for *Anemarrhena*, and green for Cork). The blue squares represent the targets of each compound. One target can have multiple compounds and vice versa

**Fig. 4:**
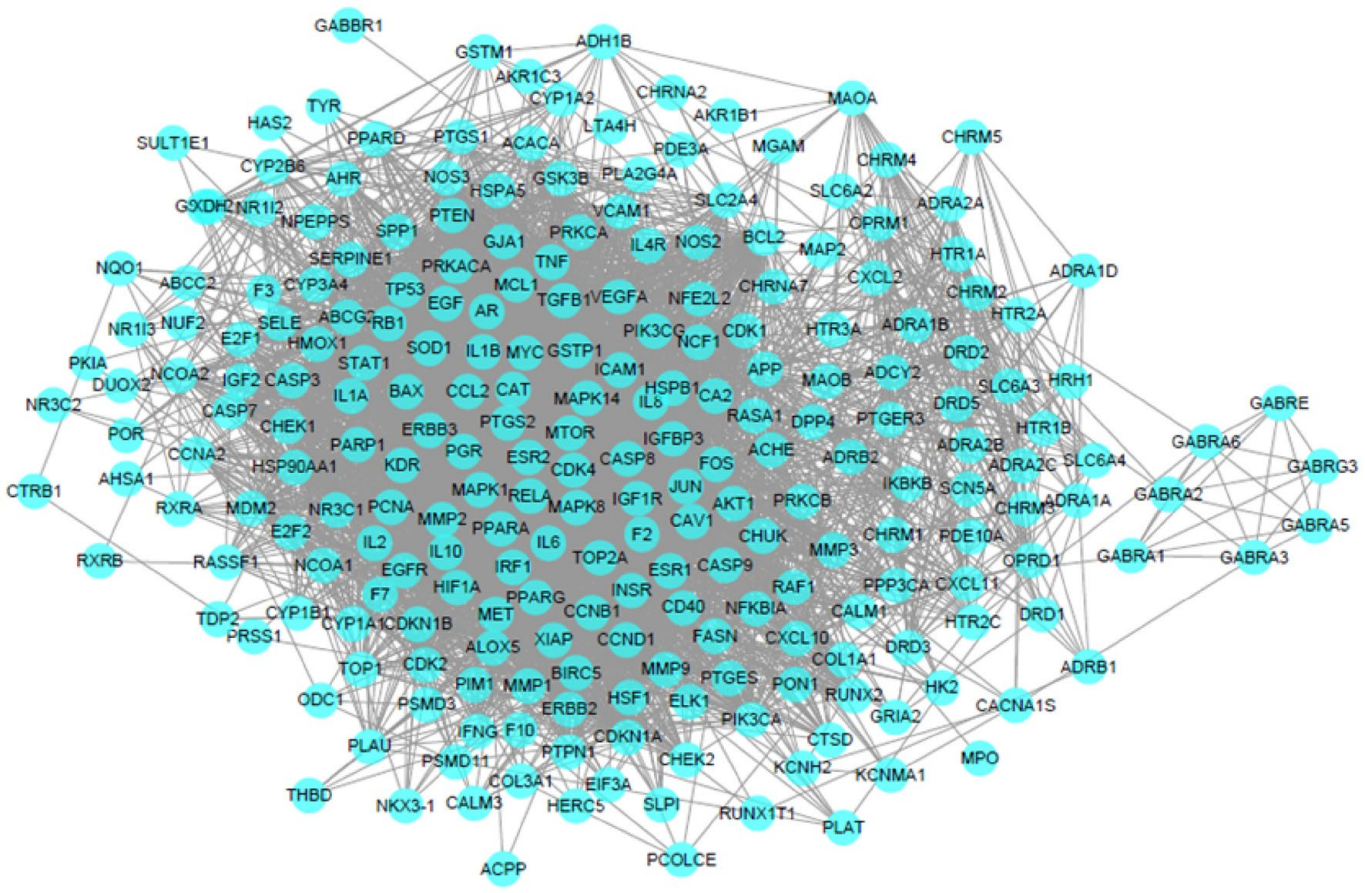
PPI-target interaction network. Nodes represent targets, proteins, and genes of EXD. The edges represent the links between nodes. There are 238 nodes and 3880 edges in this network

**Fig. 5:**
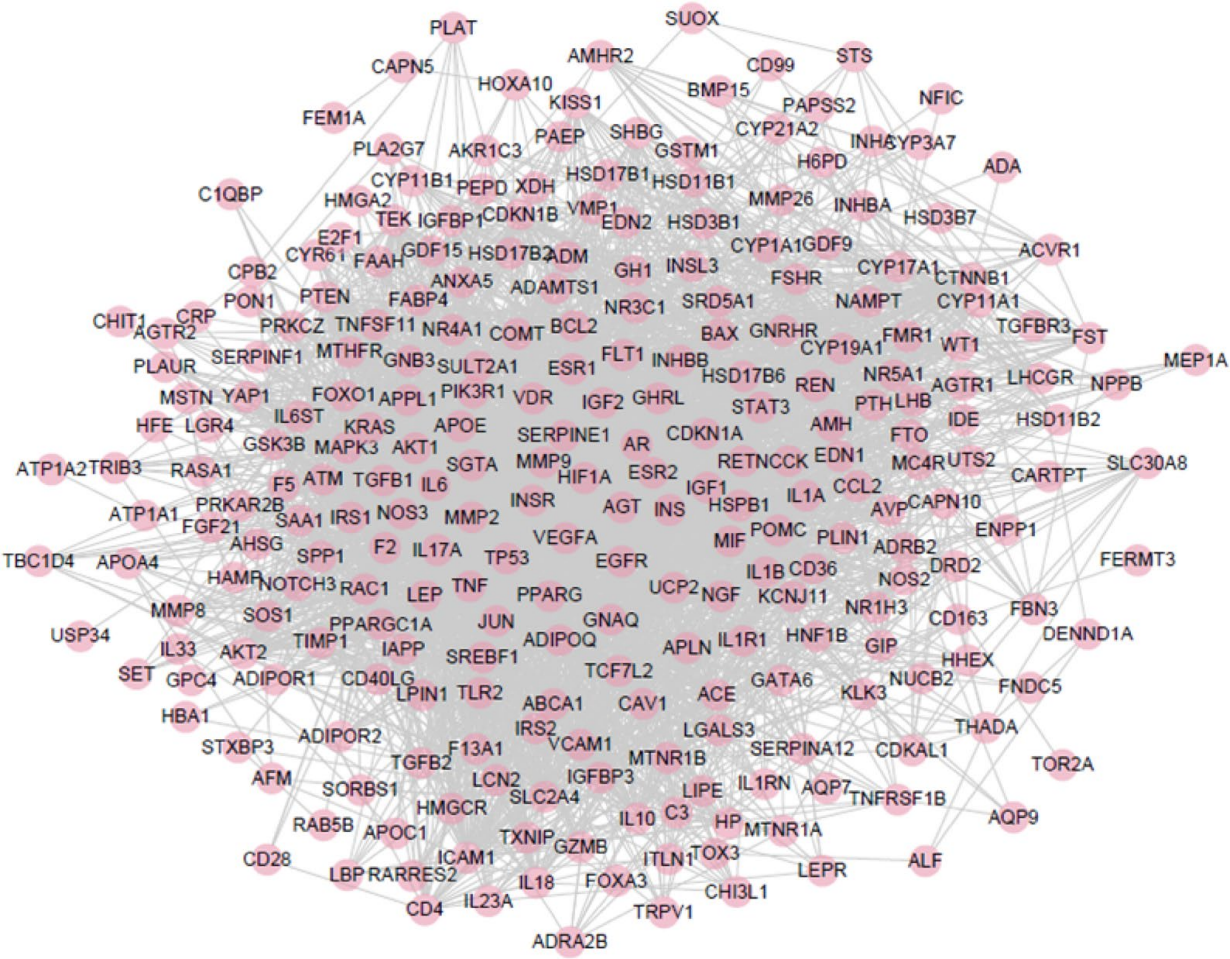
A PPI interaction network of PCOS-related genes. Dots represent genes associated with PCOS, and edges represent interactions between genes. There are 262 nodes and 3428 edges in this network

**Fig. 6:**
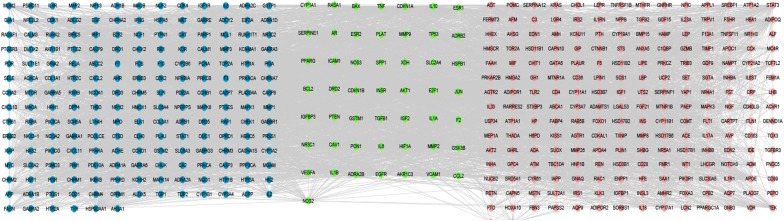
PCOS-related drug targets. Pink nodes represent genes associated with PCOS, blue nodes represent EXD targets, green nodes represent co-acting genes, and the edges represent inter-nodal relationships

To determine the relationship between the active compounds of EXD with their putative targets, a compound-target (CT) network was first built (Fig. 3). In such a network, nodes with degree greater than twice the median are considered key nodes; accordingly, 71 hub targets and 13 central compounds were obtained. The protein–protein interaction (PPI) network was constructed using the 247 drug targets, which revealed 238 nodes and 3880 edges. The network radius, diameter and characteristic path length were 3, 5 and 2.2, respectively. The greatest degree was of AKT1 (125), followed by JUN (121), TP53 (118), FOS (113), and EGFR (101). A total of 58 central targets were obtained that had values greater than twice the median (Fig. 4). In addition, the potential targets associated with PCOS were retrieved from the NCBI Gene database, and a network of related genes was built using STITCH, which showed 262 nodes and 3428 edges (Fig. 5). We mapped the drug interaction network to the PCOS-related gene interactions network to obtain the drug-PCOS interaction network (Fig. 6). Pink nodes represent genes associated with PCOS, blue nodes represent EXD targets, green nodes represent co-acting genes, and the edges between nodes represent inter-nodal relationships. A total of 50 targets were identified which are likely the key drug targets in PCOS.

### Biological functional analysis

**Fig. 7:**
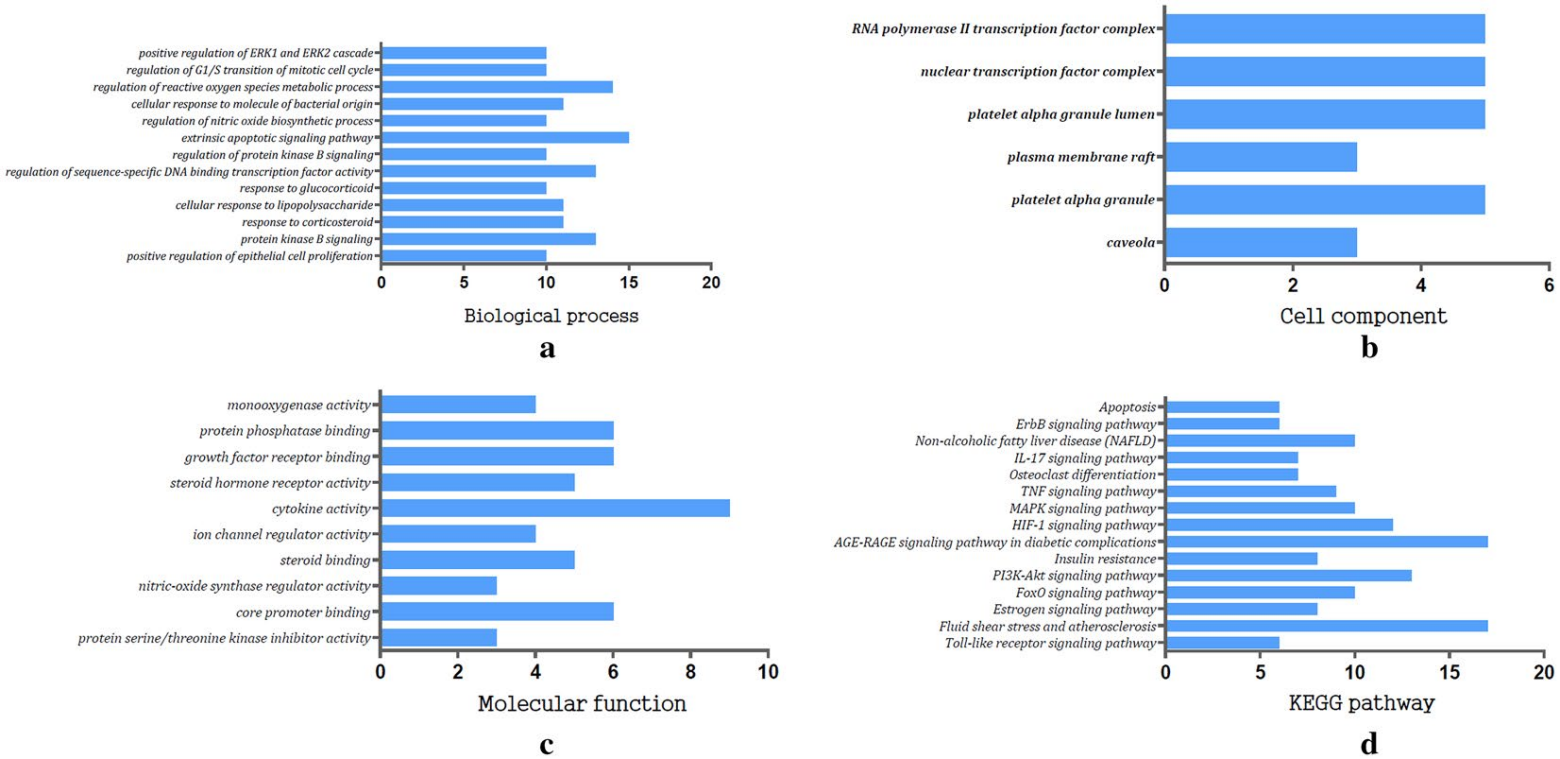
GO functional analysis. **a** Biological processes terms were extrinsic apoptotic signaling pathway, positive regulation of reactive oxygen species metabolic process, protein kinase B signaling, positive regulation of sequence-specific DNA binding transcription factor activity, and response to corticosteroids etc. **b** Cell component terms included plasma membrane raft, nuclear transcription factor complex, caveola RNA polymerase II transcription factor complex, platelet alpha granule, platelet alpha granule lumen etc. **c** Molecular function terms were growth factor receptor binding, cytokine activity, ion channel regulator activity, nitric-oxide synthase regulator activity, steroid binding etc. **d** Significant KEGG pathways were AGE-RAGE in diabetic complications, fluid shear stress, atherosclerosis, PI3K-Akt, MAPK, and FoxO signaling pathway etc

**Fig. 8:**
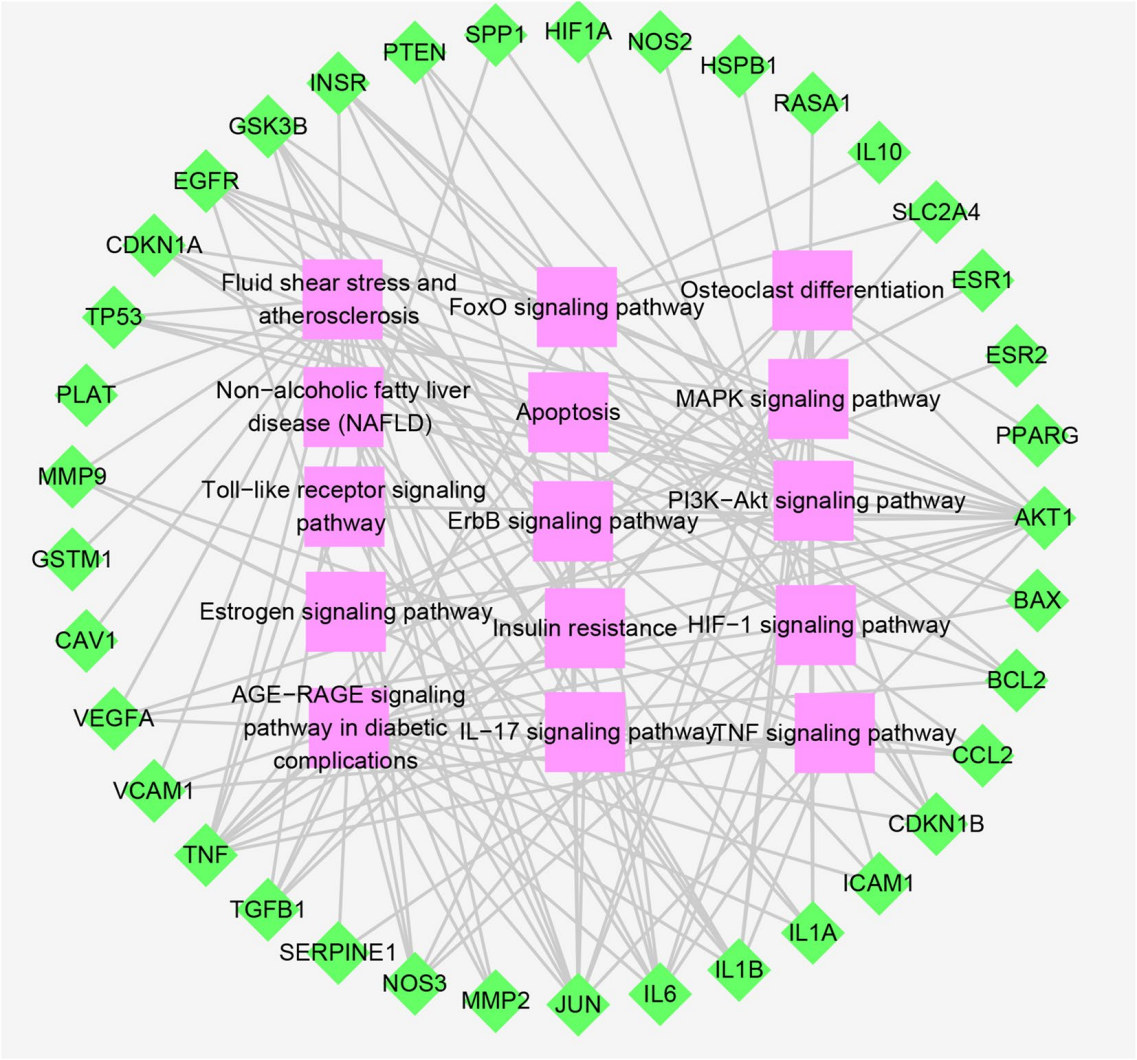
Analysis of Biological Function of KEGG Pathways (Pathway-Target network). The green points represent the key targets of PCOS relevant to EXD action, and the pink points represent the path of action related to the targets

Biological functions of the PCOS specific drug targets were annotated to clarify the mechanism of action of EXD in PCOS. GO enrichment analysis was performed on the 50 targets using ClueGO, and the top five BP terms were extrinsic apoptotic signaling pathway, positive regulation of reactive oxygen species metabolic process, protein kinase B signaling, positive regulation of sequence-specific DNA binding transcription factor activity, and response to corticosteroids. The top five MF terms were growth factor receptor binding, cytokine activity, ion channel regulator activity, nitric-oxide synthase regulator activity and steroid binding, while the main CC terms were plasma membrane raft, nuclear transcription factor complex, caveola RNA polymerase II transcription factor complex, platelet alpha granule and platelet alpha granule lumen (Fig. 7). The significant KEGG pathways were AGE-RAGE in diabetic complications, fluid shear stress and atherosclerosis, PI3K-Akt, MAPK and FoxO, among a total of 15 pathways (Fig. 8).

## Discussion

Traditional Chinese medicine (TCM) consists of complex formulations which have hitherto been difficult to characterize, thus limiting their widespread clinical use. In this study, we combined systems pharmacology (SP), pharmacokinetics (PK) and bioinformatics to identify the individual compounds of the EXD formula and their specific PCOS-related targets. EXD is a formulation of six herbs with known ameliorative effects in gynecopathy.

After screening EXD for OB and DL, 70 compounds were obtained, of which the most effective were C28 (quercetin, 153 targets) and C13 (kaempferol, 63 targets). Two constituent herbs of EXD contain these compounds. Quercetin, a polyphenol derived from many plants species, is known for its anti-carcinogenic, anti-inflammatory and antiviral activities, in addition to its active roles in platelet aggregation, lipid peroxidation and capillary permeability [37], cellular uptake, and free radical quenching [38]. Rezvan et al. found that oral quercetin supplementation enhanced AMPK levels to improve the metabolic features of PCOS in a randomized placebo-controlled double-blind trial [39]. Wang et al. showed that quercetin inhibited the Toll-like receptor/NF-kB signaling pathway and improved the inflammatory microenvironment of the ovarian tissue in a PCOS rat model [40]. Kaempferol, a dietary flavonoid, has antioxidant, anti-inflammatory, anti-apoptotic, anticancer, estrogenic, and anti-estrogenic activities [41]. It regulates the MAPK pathway to protect against IR injury by attenuating inflammation and apoptosis [42]. Nevertheless, since each herb of EXD contains multiple compounds, they act on multiple targets via several mechanisms.

The drug target network and the PCOS network had 50 overlapping genes, of which 37 were enriched in 15 pathways, and are likely the key genes involved in PCOS treatment. The significant pathways involving the candidate compounds targeting PCOS can be classified as prototypes, direct and indirect pathways depending on their functions. Based on the network analysis, we obtained three proteins of interest—AKT1, IL6 and INSR. In the hyper-androgenic PCOS patients, high levels of AKT1 have been associated with GCs dysfunction [43]. In addition, IL6, IL1B1 and TNF are associated with increased susceptibility to PCOS [44], and INSR plays a role in compensatory hyperinsulinemia [45]. EXD can regulate the expression of these genes via the AGE-RAGE, PI3K-Akt, and MAPK signaling pathways. The PI3K-Akt signaling pathway [46], non-alcoholic fatty liver disease (NAFLD) [47], MAPK signaling pathway [48], FoxO signaling pathway [6], insulin resistance [4], apoptosis, and Toll-like receptor signaling pathway [46] are strongly correlated with the occurrence and development of PCOS. Therefore, they are mechanistically important for PCOS and may play a role in its treatment as well. Studies show that one pathway contains multiple targets, and each target can act on the multiple pathways, thereby creating an intricate network.

## Conclusions

Systems pharmacology and genomics were combined to identify the PCOS relevant targets of the EXD formula. Our findings indicate that 6 of the constituent herbs in EXD act synergistically on certain putative PCOS targets. This study presents a high throughput and economic method of identifying drug targets, and may have significant clinical utility.

## Abbreviations

PCOS: polycystic ovary syndrome
PCOM: polycystic ovarian morphology
EXD: Erxian decoction
TCMSP: traditional Chinese medicine system pharmacology analysis platform
GO: Biological Network Gene Ontology tools
KEGG: Kyoto Encyclopedia of Genes and Genomes
TCM: traditional Chinese medicine
CC: cellular components
MF: molecular functions
BP: biological processes
FSH: follicle stimulating hormone
LH: luteinizing hormone
E2: estradiol
P: progesterone
MDA: malonic dialdehyde
T-AOC: total anti-oxidative capacity
ADME: absorption, distribution, metabolism, excretion
OB: oral bioavailability
DL: drug-likeness
NAFLD: non-alcoholic fatty liver disease
